# Metabolic risk factors in mice divergently selected for BMR fed high fat and high carb diets

**DOI:** 10.1371/journal.pone.0172892

**Published:** 2017-02-24

**Authors:** Julita Sadowska, Andrzej K. Gębczyński, Marek Konarzewski

**Affiliations:** Institute of Biology, University of Białystok, Białystok, Poland; Hospital Infantil Universitario Nino Jesus, SPAIN

## Abstract

Factors affecting contribution of spontaneous physical activity (SPA; activity associated with everyday tasks) to energy balance of humans are not well understood, as it is not clear whether low activity is related to dietary habits, precedes obesity or is a result of thereof. In particular, human studies on SPA and basal metabolic rates (BMR, accounting for >50% of human energy budget) and their associations with diet composition, metabolic thrift and obesity are equivocal. To clarify these ambiguities we used a unique animal model—mice selected for divergent BMR rates (the H-BMR and L-BMR line type) presenting a 50% between-line type difference in the primary selected trait. Males of each line type were divided into three groups and fed either a high fat, high carb or a control diet. They then spent 4 months in individual cages under conditions emulating human “sedentary lifestyle”, with SPA followed every month and measurements of metabolic risk indicators (body fat mass %, blood lipid profile, fasting blood glucose levels and oxidative damage in the livers, kidneys and hearts) taken at the end of study. Mice with genetically determined high BMR assimilated more energy and had higher SPA irrespective of type of diet. H-BMR individuals were characterized by lower dry body fat mass %, better lipid profile and lower fasting blood glucose levels, but higher oxidative damage in the livers and hearts. Genetically determined high BMR may be a protective factor against diet-induced obesity and most of the metabolic syndrome indicators. Elevated spontaneous activity is correlated with high BMR, and constitutes an important factor affecting individual capability to sustain energy balance even under energy dense diets.

## Introduction

Obesity and its concomitant health complications have become a growing epidemic in highly developed countries all over the world, and questions concerning its origin, causes and possible remedies remain burning [1, 2, 3, 4]. The most likely explanation to date seems to be an environment by gene interaction [4, 5, 6, 7, 8, 9, 10]. While sedentary lifestyle and access to calorie-dense diet are easily identified as the obesogenic environment, the exact genetic makeup predisposing some to gain excesses weight and develop metabolic disorders seems to be a more complex problem. Rather than a single or a fixed set of genes, obesity is most likely a product of combination of multiple genes underlying different physiological as well as behavioral traits [11, 12]. They can be categorized into basic groups related to metabolic rate and thermogenic efficiency, physical activity, appetite regulation, adipocyte storage capacity and lipid oxidation capacity [12].

The notion that low basal metabolic rates (BMR) may be associated with metabolic thrift and obesity has been evoked in literature several times. BMR is a widely accepted measure of energy expenditure in post-absorptive, resting endothermic organisms within their thermoneutral conditions [13], and essentially represents minimal cost of living. BMR is also known to be highly variable trait even among individuals of the same species and similar size, as it depends strongly on factors like body composition [14]. Lower BMR equals lower basic maintenance costs, therefore in conditions of energy dense food abundance and limited physical activity it is reasonable to expect that an energy imbalance would be easily created, leading to excessive fat accumulation and eventually—obesity. Considering that BMR accounts for approximately 50% of the human daily energy expenditure it becomes of ample importance in sustaining energy balance, with even the smallest variation being potentially critical [15, 16].

Several studies have claimed to identify low BMR to be a risk factor in weight gain in humans [17, 18, 19, 20, 21, 22]. The major problem with most of these is that they usually focus only on already obese, obese-diabetic and formerly obese individuals, which by default due to their body composition (low lean-high fat mass) show lower BMR. Another problem concerns physical activity, specifically spontaneous physical activity (SPA; i.e. associated with everyday tasks) which role in maintaining energy balance is often understated in human studies, it is also not clear whether low activity precedes obesity or is a result of thereof. SPA is a heritable trait, with a complex relationship with other physiological traits including BMR or aerobic capacity [23, 24, 25]. SPA is also known to display some plasticity in response to changing energy balance (i.e. downregulation in calorie restricted subjects, increase in overfed individuals) [26, 27, 28, 29, 30, 31, 32]. The choice of subjects, limited background knowledge and often underestimated activity records in human studies make it hard to assess the true impact of BMR and SPA on susceptibility to obesity.

Since BMR is a trait determined by multiple gens, there is no way to create a knock-out type animal model. There is however a possibility of creating a model by means of artificial selection and quantitative genetics, that would allow one to achieve a trait variability high enough for unambiguous testing of the links between innate BMR, correlated traits and susceptibility to obesity. In this study we used a unique model- mice selected for divergent BMR rates, characterized by a 50% between-line type difference (equivalent to 8.6 phenotypic standard deviations) in the primary selected trait to investigate the putative link between BMR, SPA and the diet related propensity to metabolic risk factors, particularly susceptibility to obesity. Although the selection line types are not replicated, differences in BMR, as well as SPA have been consistently shown to result from the applied selection protocol [25, 30, 33], making them an ideal model for testing various mechanistic scenarios and physiological correlations.

Here we fed the high/low BMR selected mice a high fat or a high carbohydrate diet for four months, and monitored their energy assimilation and SPA. We also analysed their body fat along with several basic biochemical parameters commonly associated with obesity related health issues (e.g. blood lipid profile, glucose), as well as oxidative stress, which can be elevated due to the pro-inflammatory fat tissue activity, or is associated with higher energy expenditures and physical activity [34, 35, 36]. Since mice with genetically determined high BMR tend to have higher SPA [30] we expected them to present the obesity- resistant spendthrift phenotype when fed the obesogenic diets, manifested by lower body fat, better lipid profile and lower blood glucose levels.

## Materials and methods

### Animals and experimental setup

All procedures were approved by the by the Local Ethical Committee on Testing Animals, Medical University of Białystok, Poland (permit no. 42/2011, 11/2013, 21/2013).

We used 120 male mice originating from the 41 generation of a long-term selection for high and low basal metabolic rate (BMR) carried out in the Institute of Biology, University of Białystok. The imposed selective regimen is designed to produce two line types of animals differing with respect to body mass-corrected BMR (high, H-BMR and low, L-BMR line type; for details see Gębczyński and Konarzewski, 2009; Sadowska et al. 2015). In each generation we maintain 30–35 families depending on the current reproductive success in the selection line types. BMR measurements are carried out when the animals are 12 weeks old. BMR is measured as oxygen consumption by means of flow-through respirometry during 2 h of a 3 h trial. Before the measurement animals are fasted for approximately 3 h to eliminate the costs of food digestion, and the measurement is performed at 32°C, within the thermoneutral zone for mice (for details on metabolic measurements see Gębczyński and Konarzewski, 2009; Sadowska et al. 2015). After BMR measurements, no more than three individuals per family with the highest (for the H-BMR line type) and lowest (for L-BMR line type) BMR scores are chosen as progenitors for further selection.

For this experiment mice were randomly divided into three groups, each consisting of 20 H-BMR and 20 L-BMR type males (all mice in a given group came from different families). Each group was then randomly assigned to one of the following diets: high fat (HFat; Labofeed, Kcynia, Poland), high carbohydrate (HCarb; Labofeed, Kcynia, Poland) or a standard control (C; Labofeed, Kcynia, Poland; for detailed composition of diets see S1 Table). Animals were then subjected to the respective diet regimens for the following 16 weeks of the experiment in standard housing conditions (23°C, 12L:12D). For mice housed individually a temperature falling within the range of 23–25°C is a standard for mimicking human physiology [37]. During the experiment all animals were housed individually with sawdust bedding and ad libitum access to food and water. Body mass was recorded weekly.

### Energy assimilation from food

At the end of first and last month of the experiment we measured energy assimilation from food. We placed animals in cages without bedding and equipped with plastic grids for a 2 day period. Food remains were collected from the bottom of the cage and separated from feces, then dried in an oven at 65°C, and weighed to the nearest 0.01 g. Average food intake was calculated individually for each mouse during the 2-day trials as the mass of food disappearing from the food dispenser minus orts. Caloric value of food and feces samples was estimated by oxygen bomb calorimetry (IKA Werke 7000 calorimeter, Germany). Daily energy assimilation was calculated for each animal as follows: ((mass of food consumed × caloric value of food)–(mass of feces × caloric value of feces))/2.

### Activity measurements

Spontaneous activity was measured 4 times (in the final weeks of 1, 2, 3 and 4 month) throughout the experiment. Unfortunately, due to hardware malfunction data from the 4th measurement were lost. For the measurements we used passive infrared sensors (TL-xpress, Crow Electronics Engineering, Fort Lee, NJ, USA) installed over each cage and monitored each 1 s by a computer (USB-6501, National Instruments, Austin, TX, USA). The entire measurement period lasted 2 full days, but readings taken for the analysis comprised an 24 hour period (from midnight to midnight) undisturbed by handling of animals. We analysed each animal’s activity in three ways: (1) as a total SPA, calculated as the daily sum of all active periods, which is analogous to the measure used in an earlier study [30], (2) the duration of SPA, calculated as the sum of 1-minute intervals, with any SPA recorded; and (3) SPA intensity representing the average amount of activity per minute when any home-cage activity was occurring (calculated as total SPA divided by the duration of SPA). Total SPA is a representation of all energy expended on spontaneous physical activity, and is a product of the duration of SPA (duration of the active phase) and intensity of active movements during this phase (SPA intensity). Both components of SPA were shown to be significant predictors of variation in food intake in Swiss mice [38].

### Blood and tissue sample analysis

In the week preceding the conclusion of the experiment fasting blood glucose levels were measured using blood glucose test strips (Optium Xido, Abbott, UK). The tail vein was punctured with a sterile needle and a blood droplet was used immediately to perform the test. At the end of the final week of the experiment blood was collected via orbital sinus puncture and immediately centrifuged. Collected serum was stored at -80°C until analysed. Total cholesterol, HDL and LDL/VLDL cholesterol fractions and triglyceride blood concentration were measured with the BioAssay Systems HDL and LDL/VLDL Assay Kit (E2HL-100) and BioAssay Systems Triglyceride Assay Kit (ETGA-200), respectively. Immediately afterwards animals were killed and dissected. All metabolically active organs were collected and weighted. Tissue samples were immediately frozen in liquid nitrogen and stored at -80°C, along with carcasses, for later body fat analysis. Organ samples (liver, kidney, heart) were analysed for oxidative damage (lipid peroxidation) by means of the TBARS method, which measures malondialdehyde (MDA) formation [39]. Protein content was also determined for each organ by the Lowry method [40] with Peterson modification [41], and the results were expressed as MDA nmol mg^-1^ protein.

### Body fat analysis

Thawed carcasses were dried at 65°C to a constant mass and then homogenized with an electric mill. Fat was extracted from weighted homogenate samples with petroleum ether in a Soxhlet extractor. The residues remaining after extraction were then re-dried, and the fat content was calculated as the mass lost during extraction [42].

### Statistics

BMR and body fat were analysed by means of ANCOVA with the line type and diet as fixed factors, and body mass as a covariate. In cases where line type × diet interaction was significant data were analysed separately for each line type. We analysed body mass gain rate (expressed as regression coefficients of individual mass gain for weeks 2–16) with ANOVA, with line type and diet as fixed factors. Likewise, we used same-structured ANOVA to analyse differences in body mass at the beginning of the experiment, as well as blood parameters (glucose, triglyceride, cholesterol level) at its conclusion.

SPA was analysed by means of repeated-measures ANOVA with the line affiliation and diet as the between-subjects fixed factors, and the time course as the within-subjects independent factor, along with their interactions. The effect of the particular infrared sensor was included as random factor. Energy assimilation was analysed with repeated measures ANCOVA with line type and diet as fixed factors, and body mass as a covariate. Post hoc comparisons were performed with a Tukey’s test. We also used the above described models to approximate the proportion of total variance of body fat and SPA explained by the line type affiliation and diet. For this we used the ratio of the type III sum od squares of the respective effects to the total sum of squares.

All statistical analyses were carried out with SAS 9.3 software (SAS Institute, Cary NC, USA).

## Results

### Energy assimilation

The line type × diet × month of measurement interaction was significant (ANCOVA, F_7,195_ = 2.15; P = 0.040), and therefore we analysed data on energy assimilation separately for each month of study. We found that the effect of line- type affiliation was at the verge of significance in the first, and significant in the fourth month, while the diet effect was apparent in both the first and fourth month, with assimilation of HCarb and Control diets higher than that of HFat in the first month, and Control diet lowest in the final 4^th^ month (Table 1). Effect of body mass was not significant (Table 1).

**Table 1:** ANCOVA results and mean values for energy assimilation (least square means ±s.e.m) measured in the first and fourth month of the experiment in H-BMR and L-BMR type mice fed a HCarb, HFat and Control diet. Significant between-diet group differences are marked by different letters (a,b).

Month	Line type	Diet	Body mass	Line type × diet		H-BMR (kJ day^-1^)	L-BMR (kJ day^-1^)	Between-diet differences
1	F_1,102_ = 3.70	F_2,102_ = 7.74	F_1,102_ = 0.63	F_2,102_ = 0.35	HFat	75.90 ± 2.89	68.61 ± 2.84	a
	P = 0.057	P = 0.001	P = 0.430	P = 0.708	HCarb	87.78 ± 2.97	81.27 ± 2.92	b
					Control	80.86 ± 2.91	78.55 ± 3.11	b
4	F_1,92_ = 11.97	F_2,92_ = 6.77	F_1,92_ = 2.62	F_2,92_ = 0.80	HFat	72.49 ± 2.92	60.44 ± 3.41	a
	P = 0.001	P = 0.001	P = 0.108	P = 0.451	HCarb	71.94 ± 2.84	66.36 ± 2.95	a
					Control	62.62 ± 3.31	55.96 ± 3.27	b

### Spontaneous activity

**Fig 1:**
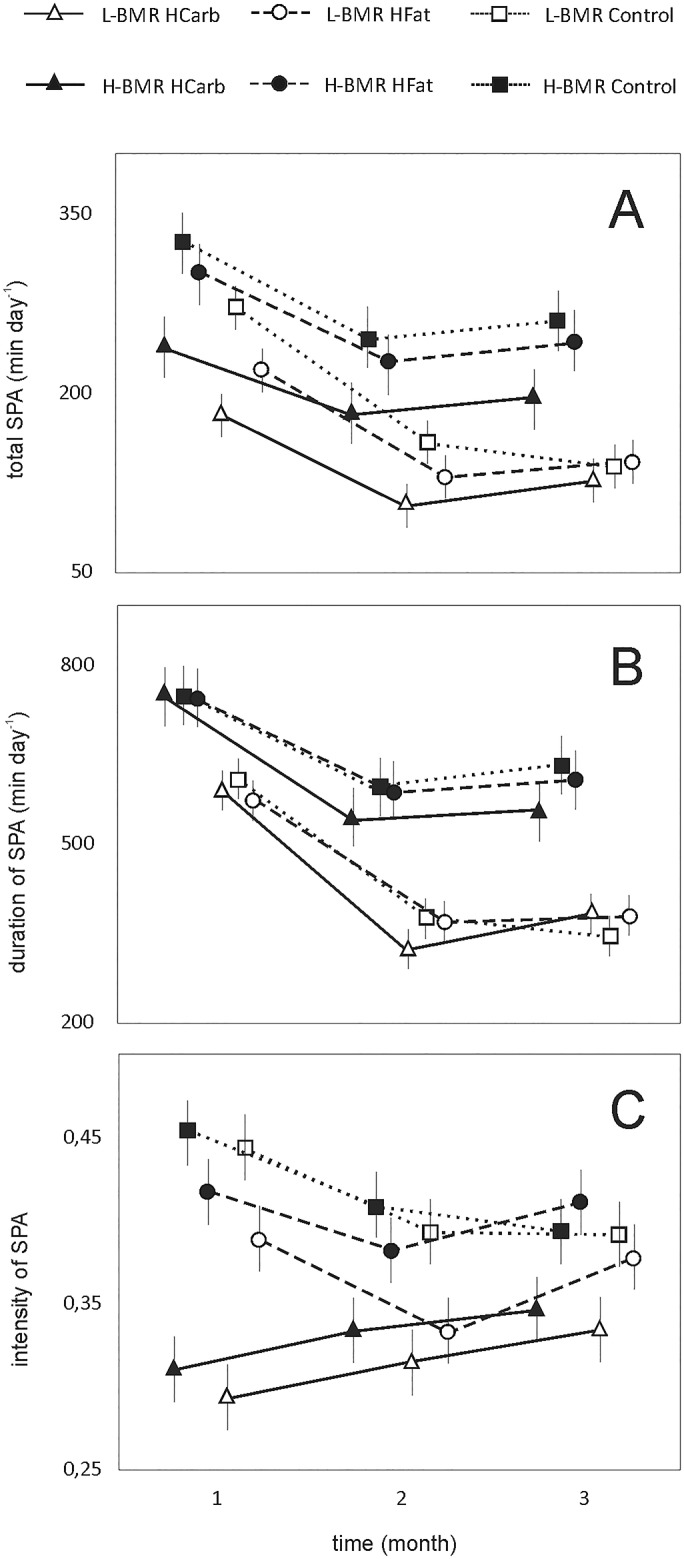
Total SPA (A), SPA duration (B), and SPA intensity (C) in H-BMR and L-BMR mice subjected to three diet regimens.

Total SPA was consistently higher in the H-BMR line type (Fig 1). There was also a significant effect of diet, with the highest total SPA recorded in the Control group (Table 2; Fig 1). Total SPA was also highest in the first month (Table 2; Fig 1). Line type affiliation explained respectively 3.6%, 14.3% and 9.1% of total SPA variation in the 1^st^, 2^nd^ and 3^rd^ month, whereas diet accounted for: 23.4%, 8.0% and 6.1% of SPA variation, respectively.

**Table 2:** Repeated measures ANOVA results of all three estimates of SPA recorded at the end of the experimental months in H-BMR and L-BMR mice fed a HFat, HCarb and Control diet.

	Total SPA	Duration of SPA	Intensity of SPA
Line type	F_1,306_ = 46.54	F_1,306_ = 52.10	F_1,306_ = 6.85
	P < 0.001	P < 0.001	P = 0.009
Diet	F_2, 306_ = 24.97	F_2,306_ = 1.65	F_2,306_ = 42.37
	P < 0.001	P = 0.193	P < 0.001
Month of measurement	F_2,306_ = 49.69	F_2,306_ = 86.07	F_2,306_ = 2.09
	P < 0.001	P < 0.001	P = 0.125
Line type × diet × month of measurement	F_12,306_ = 1.19	F_12,306_ = 0.99	F_12,306_ = 2.09
	P = 0.286	P = 0.456	P = 0.017

Duration of SPA was also significantly longer in the H-BMR type mice, with the highest scores recorded in the first month (Table 2; Fig 1). Here, however, we found no effect of the diet (Table 2; Fig 1), which points to SPA intensity as the major component accounting for the between diet differences in total SPA. Line type affiliation accounted for 4.5%, 22.5% and 11.8% of the SPA duration variation in the respective three measurements, while diet explained 1.4%, 2.9% and 1% respectively.

The line type × diet × month of measurement interaction significantly affected the SPA intensity (Table 2). Therefore, we analysed the data for the three months separately. In none of the three months SPA intensity showed between-line type differences (1^st^: F_1,111_ = 2.45; P = 0.120; 2^nd^: F_1,111_ = 2.57; P = 0.112; 3^rd^: F_1,112_ = 0.77; P = 0.381; with line affiliation explaining respectively 0.2%, 2.1% and 1.2% SPA intensity variation). The diet effect was however significant in all consecutive months (1^st^: F_2,111_ = 50.91; P < 0.001; 2^nd^: F_2,112_ = 6.51; P = 0,002; 3^rd^: F_2,112_ = 3.57; P = 0.031 and accounted, respectively, for 43.2%, 9.5% and 8.9% of SPA intensity variation), with the HCarb lowering SPA intensity in a similar fashion in both line types.

### Body and dry fat mass

Initial body mass did not differ between the two line types (F_1,108_ = 1.36; P = 0.246) nor the experimental groups (F_2,108_ = 0.53; P = 0.589). Body mass gain rate differed between the line types (F_1,101_ = 12.72; P = 0.001) with the L-BMR mice gaining weight faster. Diet did not affect the weight gain (F_2,101_ = 0.35; P = 0.708), there was also no line type × diet interaction (F_2,101_ = 1.17; P = 0.315).

**Fig 2:**
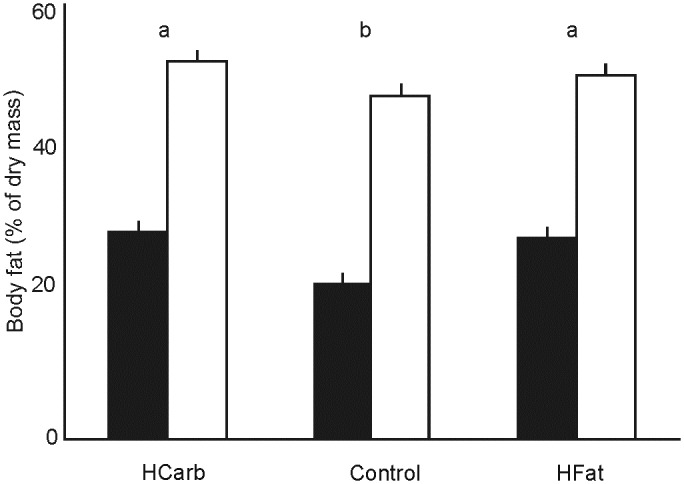
Dry mass body fat content (%) in H-BMR (black bars) and L-BMR (white bars) mice fed the HFat, HCarb and Control diet. Data presented as least square means ± s.e.m. Different letters (a,b) show significant between-diet group differences.

Dry body mass fat percentage was significantly affected by the line type affiliation (F_1,31_ = 197.05; P < 0.001) with the L-BMR type mice showing higher fat content. There were also between-diet differences (F_2,31_ = 11.58; P = 0.001) with mice fed the Control chow having lower body mass fat percentage (Fig 2). We also found that body mass affected fat mass (F_74,31_ = 2.27; P = 0.006) with no line type × diet interaction (F_2,31_ = 1.61; P = 0.327). Line type affiliation, diet and body mass explained respectively 8%, 0.54% and 7% of dry body fat mass variation.

### Oxidative damage

MDA concentration was significantly higher in the livers and hearts of the H-BMR animals (liver: F_1,106_ = 7.44; P = 0.007; heart: F_1,105_ = 6.50; P = 0.012; Table 3), however with no effect of the diet (liver: F_2,106_ = 1.16; P = 0.316; heart: F_2,105_ = 0.06; P = 0.944). In kidneys the MDA concentrations did not differ between the two selection line types (F_1,106_ = 0.69; P = 0.406) and diets (F_2,106_ = 0.45; P = 0.641). In all cases there were no interactions (liver: F_2,106_ = 1.29; P = 0.279; heart: F_2,105_ = 0.11; P = 0.897; kidneys: F_2,106_ = 1,55; P = 0.217).

**Table 3:** MDA concentration in organs of H-BMR and L-BMR type mice fed a HFat, HCarb and Control diets. Data presented as means ± s.e.m.

Diet	Line type	Kidneys (nmol mg^-1^ protein)	Heart (nmol mg^-1^ protein)	Liver (nmol mg^-1^ protein)
HCarb	H-BMR	0.452 ± 0.062	0.255 ± 0.027	0.099 ± 0.012
	L-BMR	0.530 ± 0.062	0.210 ± 0.029	0.074 ± 0.012
HFat	H-BMR	0.495 ± 0.064	0.265 ± 0.029	0.076 ± 0.012
	L-BMR	0.427 ± 0.062	0.204 ± 0.028	0.068 ± 0.012
Control	H-BMR	0.590 ± 0.066	0.261 ± 0.030	0.113 ± 0.013
	L-BMR	0.451 ± 0.061	0.190 ± 0.028	0.065 ± 0.012

### Lipid profile and blood glucose

**Fig 3:**
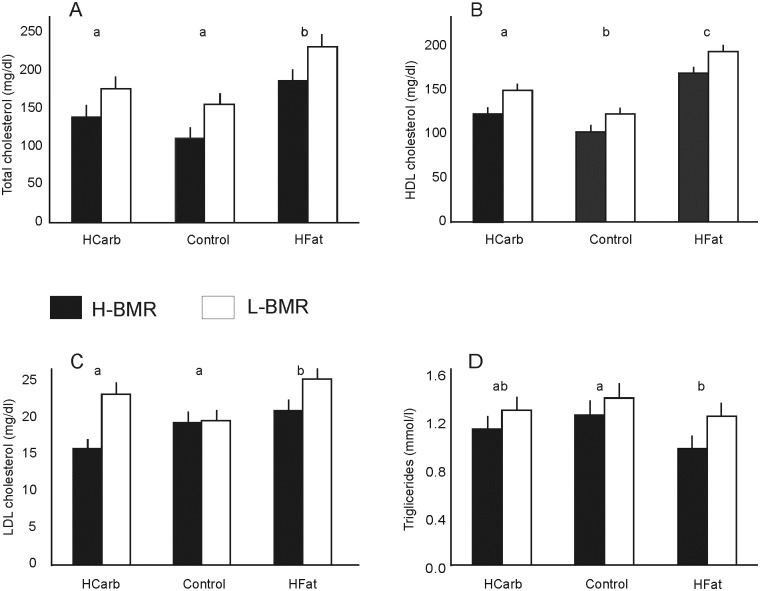
Lipid profile: total cholesterol (A), HDL cholesterol (B), LDL cholesterol (C) and triglyceride (D) blood levels in mice fed the HFat, HCarb and Control diets. Data presented as least square means ± s.e.m. Different letters (a,b,c) show significant between-diet group.

All cholesterol fractions differed significantly between the two line types (Table 4) with higher values in the L-BMR line type (Fig 3). Diet type also affected cholesterol levels with HFat diet yielding the highest values (Table 4, Fig 3). The line type × diet interaction was not significant with the exception of the LDL/VLDL fraction (Table 4). When analysed separately for each diet type the LDL/VLDL values showed significant between-line type differences only for the HCarb and HFat diets (HCarb: F_1,36_ = 11.97; P = 0.001; HFat: F_1,34_ = 4.47; P = 0.041) but not the Control (F_1,34_ = 0.01; P = 0.907), also with the HFat regimen yielding the highest cholesterol concentrations.

**Table 4:** ANOVA results of lipid profile and blood glucose of H-BMR and L-BMR type mice fed a HCarb, HFat and Control diet.

	Line type	Diet	Line type × diet
Total cholesterol	F_1,104_ = 14.80	F_2,104_ = 16.13	F_2,104_ = 0.040
	P < 0.001	P < 0.001	P = 0.965
HDL	F_1,104_ = 20.17	F_2,104_ = 56.01	F_2,104_ = 0.13
	P < 0.001	P < 0.001	P = 0.877
LDL/VLDL	F_1,104_ = 11.85	F_2,104_ = 4.37	F_2,104_ = 3.33
	P = 0.001	P = 0.015	P = 0.039
Trigliceride	F_1,106_ = 8.54	F_2,106_ = 3.77	F_2,106_ = 0.37
	P = 0.004	P = 0.026	P = 0.692
Glucose	F_1,113_ = 34.62	F_2,113_ = 7.64	F_2,113_ = 0.76
	P < 0.001	P = 0.001	P = 0.468

Triglyceride level was significantly higher in the L-BMR line type (Table 4, Fig 3). The Control and HCarb diets induced significantly higher triglyceride levels (Table 4). There was no line type × diet interaction (Table 4).

Blood glucose was affected by the line type and diet with L-BMR mice and the HCarb groups showing the highest scores (Table 4). Body mass had no effect on the fasting blood glucose level (F_1,113_ = 0.29; P = 0.589), there was also no significant line type by diet interaction (Table 4).

## Discussion

Genetically determined high levels of BMR and SPA have been suggested to be among genetic factors affecting energy balance of sedentary individuals, ultimately shaping the obesity-prone thrifty, and resistant- spendthrift phenotypes [5, 20, 26, 43]. Since BMR constitutes a substantial fraction of daily energy expenditures [12, 16], in sedentary subjects it becomes one of the main outlets for utilizing excess energy, with some studies reporting it to constitute even up to 70% of the total daily expenditures of humans [15, 16]. If however high BMR is coupled with elevated SPA, such combination would significantly increase energy expenditure and possibly create an obesity-resistant spendthrift phenotype.

In this study we used mice selected for a high BMR. These mice have been shown to have larger organs with higher mass-specific metabolic rate ([44], for review see Konarzewski and Książek 2012). Here we demonstrated that H-BMR with higher SPA showed in fact lower propensity to the detrimental effects of western type diets, including lower body fat, blood glucose and overall healthier lipid profile.

Animals used in this study are a relevant model emulating the “sedentary lifestyle”—they spend their entire life in a small cage with easy access to food and water. They do not need to forage and therefore, they might as well have evolved very little of such SPA behavior. Yet, H-BMR type mice defy this scenario, and notably, their elevated SPA is a genetically determined, correlated response to selection for high BMR [25]. It is also extremely important to note that SPA level in the L-BMR line type has not been altered compared to a randomly bred strain of Swiss Webster mice, as demonstrated in a previous study [30]. Thus the between line-type difference is due to up-regulation of SPA in the H-BMR mice.

One may argue that high BMR combined with high activity stimulates a compensatory energy intake, and may therefore open an easy path to overconsuming the energy dense, highly palatable western diet [45]. Higher food consumption in the H-BMR line type was demonstrated a number of times in a variety of different conditions and protocols including: normal housing conditions, cold exposure and reproduction [30, 46]. Importantly, H-BMR mice showed higher energy assimilation (although not statistically significant in the first month; Table 1), suggesting that a truly increased energy intake was occurring.

Although physical activity itself is known to stimulate food consumption [37, 47, 48], innate SPA has been also shown to be negatively correlated with fat mass gain and obesity later in life in rats [49]. A number of papers point out that different types of activity, its duration and intensity result in particular tissue adaptations with various benefits, ranging from lower cardiovascular and metabolic syndrome risk, to enhanced strength or aerobic performance [50, 51, 52]. Therefore apart from analyzing total SPA, we also broke it down into its components: duration and intensity, which both can be altered independently by a plethora of factors, including diet. We found that the direct selection for BMR affects mainly SPA duration, which is a representation of the daily activity phases—longer in the H-BMR mice (the line type affiliation accounting from 4.5%, to 22.5% and 11.8% of the variation in consecutive months of experiment). The diet on the other hand mainly effected SPA intensity, inducing more intense bursts of activity in the Control and HFat fed mice from both line types (Fig 1). This suggests that diet type plays only a moderate role in regulating spontaneous behavior, with rather the innate SPA levels possibly priming animals for different effectiveness of substrate utilization.

In mice selected for wheel running and fed a high fat diet, elevation of SPA and the absence of excessive weight gain seems to result from greater ability to utilize fat as muscle fuel [53]. Increased fat oxidation in response to physical exercise, whether low intensity movement or high capacity training, has been found to occur only during activity, and not impacting the overall 24h fat oxidation [54, 55]. SPA of our mice was low- intensity physical activity (comparable to walking in human subjects), however with significant differences in the duration of active phase between the two line types. The longer activity phases in the H-BMR line may be important for sustaining energy balance and may explain the overall favorable lipid profile and blood glucose in the H-BMR line type. A number of human-based studies reported a correlation between physical activity and metabolic health indicators like HDL/LDL cholesterol and triglyceride level, with even the potential of reversing detrimental diet-induced changes [52].

SPA intensity seems to have weaker impact, as it was not affected by the line type affiliation, and only modulated by the diet type, particularly by the HCarb diet (from up to 43.2% of intensity variation in the first month, to 9.5% and 8.9% in the latter two months were explained by the diet effect; Fig 1A–1C). The high-sugar diet has been shown to downregulate physical activity both in human and rodent subjects, contributing to energy imbalance [56]. Indeed, in comparison to HFat diet, the HCarb diet negatively affected SPA also in our study (Fig 1A–1C).

Due to time constrains we did not measure BMR later in the experiment, but we have already demonstrated that the between-line type divergence in BMR remains at a fairly stable and highly statistically significant level even during substantially altered physiological states, such as lactation [33], or long-term calorie restriction [44]. Moreover, as we repeatedly demonstrated, the between-line type difference in BMR results from the applied selection, rather than genetic drift [30, 33]. Therefore, even accounting for any alternations due to long-term western type diet consumption we can safely assume that that throughout the experiment, the basic metabolic expenditures were maintained at significantly higher level in the H-BMR line type, and together with SPA resulted in resistance to obesity. Due to logistical limitations we were unable to quantify SPA impact on the total daily energy expenditure of our mice and more specifically, contribution of different types of activity to SPA. For example, the feed itself, or more precisely the delivery system and pellet hardness may affect time spend on feeding, thus affecting activity measurements. Our infrared sensor system did not allow us to discriminate and quantify the time spent on feeding from other activity, therefore we cannot exclude the possibility that SPA intensity differences may be also affected by more intense nibbling on the Control pellets. However, other studies using the infrared sensors show that in fact the activity measurements performed by means of this method confirm the impact of SPA on daily energy budgets [25, 37, 57]. Moreover, those higher energy expenditures of the H-BMR mice were also associated with higher oxidative stress in their metabolically active organs—liver and heart (Table 3). Higher basic maintenance costs have been associated with elevated oxidative stress, as more intense cellular respiration generates more free radicals that cause damage to lipids, proteins and DNA [35]. More recently, also physical activity has been deemed to be one of the more significant stressors inducing oxidative stress [58]. Although this may seem counterintuitive, as exercise is traditionally associated with health benefits, even low levels of activity (including everyday activity) also cause stress and some damage [35, 56], which perhaps, was reflected in higher levels of MDA found in the H-BMR mice. At this point however, we cannot determine to what extent which of the selection results—high BMR or high SPA is responsible for the higher oxidative stress.

In conclusion, we demonstrated that high BMR is a protective factor against diet-induced obesity, and has positive effect on key indicators of the metabolic syndrome. Furthermore, we demonstrated that the ability to balance energy expenditures is coupled with high spontaneous activity correlated with high BMR, and therefore constituting a vital component of thrifty genotype. Protective effect of high BMR and SPA may however be diminished by an elevated level of oxidative stress in major metabolically active organs—liver and heart. Our study demonstrated that behavioural and physiological responses to high fat and high carb diets may render our selected mouse line types an unique model for studies on metabolic risk factors.

## Supporting information

S1 Table The HFat, HCarb and Control diet detailed composition. (DOCX)

S1 Text BMR and accounting for genetic drift. (DOCX)

S1 Dataset (XLSX)
