# Supplementary material for: Metabolic risk factors in mice divergently selected for BMR fed high fat and high carb diets
Source: PLoS One. 2017 Feb 24;12(2):e0172892. doi: 10.1371/journal.pone.0172892 (PMC5325576; doi:10.1371/journal.pone.0172892)
Supplement: S1 Table — (DOCX) [file pone.0172892.s001.docx]

S1 Table. Energy density (kJ/g), Metablizable energy (kJ/g), and digestive efficiency of the HFat, HCarb and Control diets along with their detailed composition.

|  | HFat | HCarb | Control |
| --- | --- | --- | --- |
| *Energy density kJ/g | 18,3 | 17.2 | 17.0 |
| **Metabolizable energy kJ/g  H-BMR 1^st^ month H-BMR 4^th^ month  L-BMR 1^st^ month  L-BMR 4^th^ month | 13.6 ± 0.1  13.4 ± 0.1  14.3 ± 0.1  14.3 ± 0.1 | 14.2 ± 0.1  13.1 ± 0.1  14.6 ± 0.1  14.2 ± 0.1 | 12.9 ± 0.1  12.3 ± 0.1  13.5 ± 0.1  13.0 ± 0.1 |
| ***Digestive efficiency  H-BMR 1^st^ month  H-BMR 4^th^ month  L-BMR 1^st^ month  L-BMR 4^th^ month | 0.74 ± 0.01  0.73 ± 0.01  0.78 ± 0.01  0.78 ± 0.01 | 0.82 ± 0.01  0.76 ± 0.01  0.85 ± 0.01  0.82 ± 0.01 | 0.75 ± 0.01  0.72 ± 0.01  0.79 ± 0.01  0.76 ± 0.01 |
| Dry mass g/kg | 898 | 915 | 885 |
| Raw protein g/kg | 201 | 183 | 217 |
| Raw fat g/kg | 141 | 46.2 | 49.1 |
| Carbohydrates g/kg | 507 | 639.8 | 567.9 |
| Fiber g/kg | 49 | 43.9 | 54.8 |
| Ash g/kg | 53,5 | 42.2 | 59.4 |
| Calcium g/kg | 9.35 | 13.4 | 9.57 |
| Phosphorous g/kg | 6.68 | 3.84 | 7.70 |
| Sodium g/kg | 1.91 | 1.81 | 1.97 |
| Magnesium g/kg | 2.44 | 1.17 | 2.88 |
| Potassium g/kg | 9.52 | 6.67 | 10.9 |
| Manganese mg/kg | 54.8 | 24.6 | 68.1 |
| Iodine mg/kg | 0.246 | 0.219 | 0.254 |
| Copper mg/kg | 13.9 | 10.5 | 15.9 |
| Iron mg/kg | 163 | 134 | 171 |
| Zinc mg/kg | 66.3 | 41.4 | 78.9 |
| Cobalt mg/kg | 0.292 | 0.252 | 0.313 |
| Selenium mg/kg | 0.467 | 0.305 | 0.461 |
| Vitamin A IU | 15 000 | 15 000 | 15 000 |
| Vitamin D3 IU | 1 000 | 1 000 | 1 000 |
| Vitamin E mg/kg | 92.4 | 82.3 | 91.9 |
| Vitamin K3 mg/kg | 3.00 | 3.00 | 3.00 |
| Vitamin B1 mg/kg | 8.78 | 5.72 | 10.4 |
| Vitamin B2 mg/kg | 7.94 | 6.32 | 8.18 |
| Vitamin B6 mg/kg | 13.4 | 9.45 | 13.9 |
| Vitamin B12 mg/kg | 0.052 | 0.051 | 0.052 |
| Folic acid mg/kg | 2.29 | 1.89 | 2.41 |
| Nicotinic acid mg/kg | 102 | 45.9 | 111 |
| Pantothenic acid mg/kg | 25.6 | 16.7 | 27.3 |
| Choline mg/kg | 2 037 | 1 591 | 2 172 |

*Energy density kJ g^-1^ : caloric values obtained by bomb-calorimetry measurements

**Metabolizable energy kJ g^-1^: energy density x digestive efficiency

***Digestive efficiency: (mass of food consumed × caloric value of food) – (mass of feces × caloric value of feces))/ (mass of food consumed × caloric value of food)
