# Supplementary material for: Metabolic risk factors in mice divergently selected for BMR fed high fat and high carb diets
Source: PLoS One. 2017 Feb 24;12(2):e0172892. doi: 10.1371/journal.pone.0172892 (PMC5325576; doi:10.1371/journal.pone.0172892)
Supplement: S1 Text — (DOCX) [file pone.0172892.s002.docx]

**Supporting information**

**Metabolic risk factors in mice divergently selected for BMR fed high fat and high carb diets**

Julita Sadowska, Andrzej K. Gębczyński, Marek Konarzewski

**BMR differences**

There were highly significant differences in BMR in the 41 generation between the two selection line types (F_1,56_ = 1125.75; P < 0.001; H-BMR line type: 66.24 ± 0.46 ml O_2_ h^-1^; L-BMR line type: 43.99 ± 0.47 ml O_2_ h^-1^). In both cases BMR was also significantly affected by body mass (F_1,57_ = 184.87; P < 0.001).

**Accounting for genetic drift**

Our selection line types are not replicated. Therefore, we took into account a possibility that differences in BMR and adiposity may be due to genetic drift, rather than a genuine effect of artificial selection on BMR level. To tackle this concern, we additionally analyzed BMR and body fat content according to Henderson’s guidelines (Henderson 1997; Konarzewski et al. 2005). In short, those guidelines allow to quantitatively gage the observed between- line type differences in selected traits (both primary selected trait, in our case- BMR and correlated trait- adiposity) against the expected range of differences arising solely due to random segregation of genes, which may produce spurious divergence of selected line types having nothing to do with the applied selection regimen.

First, we expressed the magnitude of separation of the high and low line type of a given trait as multiples of intra-line type phenotypic standard deviations (d*_X_*), following methods of Konarzewski *et al*. (2005). Thus expressed separation reflects the presumed effect of applied selection on divergence of selected line types. Next, we estimated the 95% confidence intervals of the maximum effect of genetic drift (thereafter called d*_drift_*) for a given d*_X_*, according to equation 16 from Henderson (1997):

$\sigma_{(dx)}=4\sqrt{(h_{X}^{2}F+1/n)}$ ,

where *h^2^* is trait heritability (for BMR *h^2^* = 0.4; Konarzewski et al., 2005; for fat mass *h^2^* = 0.42; Eisen, 1992) and F the inbreeding coefficient (F = 0.274; calculated from equation 3.5 from Falconer and Mackay (1996) for the effective population size for generation 41).

The difference d*_X_* calculated for BMR equaled 8.61 and therefore far exceed the respective boundary of the 95% confidence interval (*d*_drift_) of 1.36. Likewise, the between line-type differences in adiposity exceed those expected to arise from genetic drift alone in all three diet treatments: in the control diet group *d* = 4.41 vs. *d*_drift_ =1.50; in the HF diet group: *d* = 3.52 vs. *d*_drift_ = 1.49; in the HCarb diet group *d* =3.66 vs. *d*_drift_ =1.49. Thus, although the above estimates cannot be considered as robust as inference based on multiple replication of the selection experiment (see Swallow et al. 2009), they nevertheless strongly suggest that the between line type differences reported in our paper stemmed from the applied selection regimen, rather than genetic drift.

**References**

Swallow JG, Hayes JP, Koteja P, Garland TJr. Selection experiments and experimental evolution of performance and physiology. In: Garland, T., Jr., and M. R. Rose, eds. 2009. Experimental evolution: concepts, methods, and applications of selection experiments. University of California Press, Berkeley, California.
